# On natural history collections, digitized and not: a response to Ferro and Flick

**DOI:** 10.3897/zookeys.618.9986

**Published:** 2016-09-26

**Authors:** Derek S. Sikes, Kyle Copas, Tim Hirsch, John T. Longino, Dmitry Schigel

**Affiliations:** 1 University of Alaska Museum, 907 Yukon Drive, Fairbanks, AK 99775-6960, USA; 2 GBIF Secretariat. Universitetsparken 15, DK-2100 Copenhagen Ø, Denmark; 3 Department of Biology, University of Utah, Salt Lake City, UT 84112, USA

**Keywords:** Natural History collections, Museums, digitization, GBIF, georeferencing, data sharing

## Abstract

Ferro and Flick (2015) describe their efforts to estimate the distribution for a species of rove beetle via the study of specimens from entomological collections, and compare these results to digitally accessible open data. Their study provides an informed and accurate case study that contrasts targeted data capture with generalized public repositories of digital specimen data. However, we feel the conclusions on how global biodiversity data aggregation and publication work require clarification and correction of common misconceptions that we believe will interest those concerned with the future of natural history collections and taxonomy.

## Summary of the original statements

Ferro and Flick (2015) used a classical approach to gather distribution data for a species of rove beetle, *Thoracophorus costalis*. They borrowed specimens from 38 collections, recorded specimen data, and analyzed them with niche modeling software. They were able to show that, on average, data from at least 15 separate collections were sufficient to construct a satisfactory model. They then used data currently published through the Global Biodiversity Information Facility (GBIF) network. GBIF provided an incomplete and biased set of records that, used alone, produced poor results in species distribution modeling. Therefore, the authors argue, while online sources of data like GBIF.org may have some value, their use makes it too easy to produce low quality research. They also suggest that GBIF.org should provide more frequent and prominent notices highlighting that data may be of insufficient quality.

## Our response

We thank Ferro and Flick (2015) for raising a number of important issues regarding specimen digitization and data aggregators like GBIF.org. We take this opportunity to highlight some issues which we hope our community can work together towards resolving and add a counterpoint to Ferro and Flick’s (2015) critique of GBIF.

## Taxonomy and digitization a zero-sum game?

**Figure 1.**
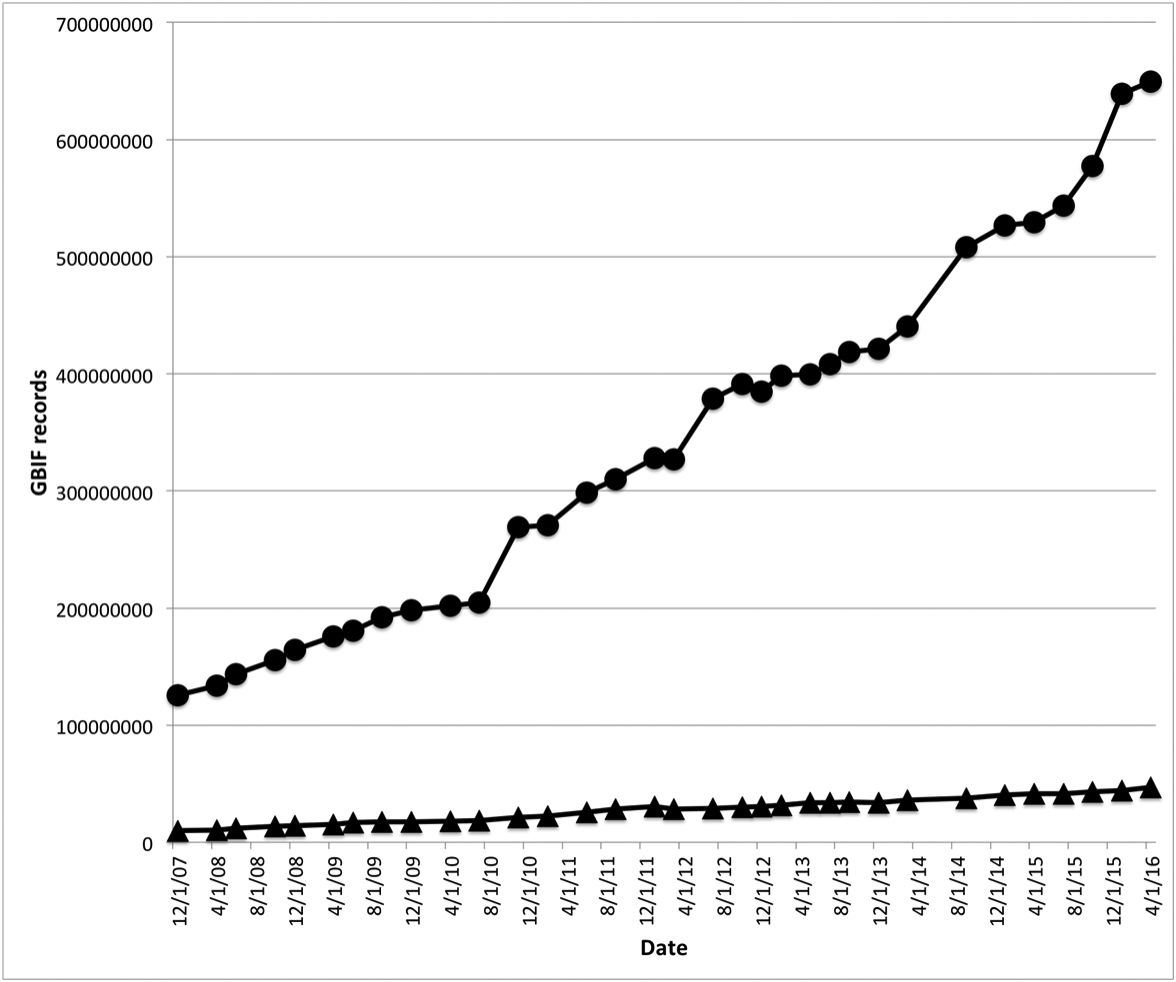
Number of insect records in GBIF.org (triangles) between December 2007 and March 2016, in comparison to all records (circles).

Ferro and Flick raise the concern that funding for digitization efforts is siphoning funds away from the maintenance of natural history collections (NHCs). We argue that the distinction between funding NHCs and the production of GBIF-mediated data is artificial – specimen records from NHCs are the foundation of the entomological data accessible through GBIF.org, with U.S. institutions alone providing 7.5 million georeferenced insect occurrence records citing a specimen as the ‘basis of record’, including 3.5 million records relating to insect specimens collected from U.S. lands (GBIF.org (2016-09-14) GBIF Occurrence Download http://doi.org/10.15468/dl.5txrti and GBIF.org (2016-09-14) GBIF Occurrence Download http://doi.org/10.15468/dl.1kayda). There need be no ‘choice’ between maintaining good regional specimen collections and the digitization and publication of data through online aggregated databases. Increasingly in the U.S. (Kaiser 2015), and the world, sharing of digitized data and published results is expected for government funded research. This is becoming such a standard that open sharing of digitized specimen data significantly increases the probability of obtaining funding for natural history collections. The choice is, therefore, becoming one of either funding NHCs and digitizing NHC data, or doing neither. The Berlin Declaration on Open Access to Knowledge in the Sciences and Humanities of 2003 (2003), promoting open access to scientific data, has been signed by 302 worldwide scientific organizations. The National Science Foundation’s Advancing the Digitization of Biodiversity Collections (ADBC) initiative is in its fifth year of implementation and has resulted in a massive mobilization of NHC data. A new five-year national initiative in the U.S., the Biodiversity Collections Network (BCoN), funded by the National Science Foundation, has been established to support the development of a sustainable community of practice that will ensure that all U.S. biodiversity collections are digitally available for research, education, informed decision-making, and other scholarly and creative activities. The vast majority of entomological specimens have yet to be digitized (Fig. 1, only 7% of the occurrence data in GBIF is entomological despite insects representing well over 75% of all species and specimens in museum collections) and doing so will take many millions of dollars and likely many decades. However, the “writing is on the wall” that the scientific and public community want NHC and taxonomic data to be digitized and freely available online, despite the challenges this entails (Maddison et al. 2012, Roche et al. 2015, Page 2016). The more high-quality NHC data become available, the more they will be used by non-taxonomists, and the more appreciation (and funding) for NHCs and taxonomy will grow.

## Digital data quality is us

We agree entirely with the sentiment Ferro and Flick promote with their quote of Soberón and Peterson (2004) “*without a strong and active taxonomic community, BI [Biodiversity Informatics] will never be more than a clever set of software tools lacking a substantial factual basis*.” However, we wish to reiterate that biodiversity data are what the taxonomic and museum community produce and are only as good as the effort applied. They are *our data* and making them available online for the scientific community strengthens the taxonomic community. Consequently, we feel it is detrimental to the taxonomic community to produce high-quality data that are not shared with aggregators like GBIF.org.

The data that Ferro and Flick (2015) downloaded from GBIF were all from the Snow Entomological Museum (SEMC, 142 records) but they also borrowed 198 specimens from SEMC and georeferenced them for modeling. Presumably they georeferenced previously georeferenced specimens (i.e. the 142 GBIF records from SEMC were possibly within the set of 198 specimens they borrowed). This raises questions about duplication of data (and hence statistical independence of data points), duplication of effort, and how best to cite online data. Ferro and Flick (2015) listed the data they obtained from GBIF (their table 1) as ‘alternative distribution data’ from GBIF. This is not an ideal way to cite online data. These data were from SEMC which should have been cited as the data provider, with a link (DOI) for the data download from GBIF. To do otherwise is to cheat the data providers of important citations for their contribution to science. We recently searched GBIF for records of *Thoracophorus costalis* (GBIF.org (31st May 2016) GBIF Occurrence Download http://doi.org/10.15468/dl.1gs48e) and note that there are now 152 records. New data were added since Ferro’s download by the Yale Peabody Museum of Natural History
(YPM) and the Essig Museum of Entomology
(EMEC) – with high-quality identifications by Ferro. Again, these data in GBIF should not be called ‘alternative distribution data,’ they should be cited as data from each NHC data provider that is shared via GBIF. A researcher is free to visit each collection’s separate website and download the data closer to the source, rather than from GBIF, but why do this? Some NHCs have data online that are not shared with GBIF. It is worth looking for these, but the different interfaces to these collection websites mean that a researcher will have to learn how to search and download data from each website separately, since each will generally have their own unique ways of presenting and organizing data. A researcher will then have to invest considerable effort converting and aggregating each dataset into a format compatible with the Darwin Core Standard (Wieczorek et al. 2009) that is otherwise shared with, and available from, GBIF.

## Taxonomy produces the highest-quality ‘dark data’

We strongly agree with Ferro and Flick (2015) in their condemnation of digitization efforts that provide no funding for curation and identification of specimens. All natural history museums have misidentified and partially identified specimens, and specimens sorted under junior synonyms (Meier and Dikow 2004, Goodwin et al. 2015 but see also Page 2015). Although the greatest digitization efforts have been to fund museums to database their holdings, the highest quality data with the fewest misidentifications comes directly from taxonomic revisionary work such as Ferro (2015). These datasets are the highest quality but sadly, most fail to be shared digitally and thus join the accumulation of what are called ‘dark data’ (Heidorn 2008). Publishing traditional ‘material examined’ lists in such taxonomic works does not fulfill the expectation of data sharing because these data are not machine-readable nor standardized for easy conversion into a format that is machine readable and often lack geocoordinates. The two most obvious solutions to this issue involve the inclusion of funding for identification verification in all digitization grants (e.g. NSF-funded programs like ADBC and iDigBio) and increased efforts to obtain and share properly-formatted datasets from taxonomists publishing research with occurrence data.

## Occurrence data sharing in taxonomy – why so rare?

As remains typical of the majority of taxonomic work currently being published, Ferro (2015) and Ferro and Flick (2015) did not share their specimen data online. The reasons for this are likely varied and include (1) a lack of tradition or expectation to do so, (2) a lack of a user-friendly data-pipeline that taxonomists can use to share and prepare their data in the best format (Darwin Core standard, Wieczorek et al. 2009), (3) a lack of motivation by journal peer reviewers and editors to encourage (or insist) that data be shared, and (4) a lack of perceived reward for doing the extra labor involved in sharing of data. Additionally, we have heard some taxonomists state they do not want to share data with GBIF because they distrust the quality of the data in GBIF. This latter point seems illogical. The data in GBIF are the data from the museums that provide data. If the data in GBIF are not to be trusted then neither are the data in the source museums. It thus seems illogical to be pro-museum and anti-GBIF.

## Data quality, mapping, and efficiency of production

Via communication with Ferro (in lit.) during which we asked about the sharing of his dataset, we learned that Ferro felt his data were not produced in a manner ideal for sharing with GBIF. Ferro also commented on the lack of a user-friendly data-pipeline to prepare and upload data to share (more on this issue below). Ferro explained that he georeferenced records using the centers of counties for each locality record rather than georeferenced them following the best practices suggestions in Chapman and Wieczorek (2006). As a result, although Ferro and Flick felt their data were of high enough quality to publish and analyze, they felt their data were not of high enough quality to share with the wider scientific community. We find this puzzling. Consequently, if we are to ever have open access to high quality digital data for the species they studied, *Thoracophorus costalis*, someone will have to georeference those 4,900+ specimens again. This will most likely be done by the staff at the NHCs which house these specimens. This is obviously not an efficient use of the limited funds available to NHCs and taxonomists. We believe, and hope most readers do too, that once data have been typed into a computer they should never need to be re-typed. By using their unique georeferencing methods certainly some time and money was saved – but was it worth it? It might be argued that sub-optimal data are better than none. Certainly Ferro and Flick (2015) thought their data were of high enough quality on which to base their analyses and publish. We are not singling Ferro and Flick (2015) out for their choice of cost-saving methods or lack of data sharing (the majority of recently published taxonomic papers that we have seen did not share their specimen data). But we do use their work and their critique of GBIF to highlight these general challenges the entire taxonomic community faces.

Distribution mapping is changing. Such maps are not constellations of the maximum number of georeferenced occurrences, but effectively projected, modeled areas where species are thought to occur with a certain uniform or changing probability. Williams et al.’s (2014) guide to the Bumble Bees of North America relied heavily on shared data for its production and is an excellent example of the use of this form of mapping. For such maps, one needs enough data to make reliable predictions, not necessarily coordinates from every known specimen, as Ferro and Flick (2015) point out. The best available – and most cost efficient – data also means not too much: at a certain level, the price of enlarging one’s dataset will continue to go up without improving the estimate of the species’ distribution. However, with less well-studied taxa like rove beetles, currently the quality of predictions generally improves with every new observation. In principle, the data accumulation curves will all flatten, even within Coleoptera (Hof and Svahlin 2015, Beck et al. 2014, Fourcade et al. 2014, García-Roselló et al. 2014).

## Data quality warnings and peer review

GBIF is currently working to improve the representation of available data to make the completeness and fitness for use of any dataset as transparent to the user as possible. We agree that GBIF.org can include clearer text and information about both the context and limitations of data accessible through GBIF. Data will always be of variable completeness and precision, and GBIF’s approach should be to ensure that users such as distribution modelers can easily restrict searches to data fit for their use, while not excluding other data that may still be useful for other purposes. However, taxonomists who are well aware that museum collections are rife with misidentifications and data quality issues such as collector bias (Hjarding et al. 2015, Goodwin et al. 2015 but see also Page 2015), should not be surprised when these issues are present in data aggregated by GBIF. Should all museums post similar warnings inside their collections? Of course, online data are available to a much wider and less well-trained user audience than physical specimens in NHCs. Thus, we agree it is wise to warn naïve users of potential data quality issues.

Indeed, not all scientific users understand that globally aggregated data always need filtering and post processing, as well as dealing with data gaps. A constructive alliance would enlist experts to help address quality issues in the process of global data aggregation. For example, despite the increasing fraction of wrongly annotated fungal sequences in GenBank, the trustworthy ones (Nilsson et al. 2012; Hyde et al. 2013) are dynamically reflected in the UNITE database (Kõljalg et al. 2013). From the UNITE webpage: “*We aim at including only high-quality sequences of well identified fungi, hence initially sacrificing quantity for quality*.”

The issue of data quality will never, and should never, go away. All data need vetting. The study of Hjarding et al. (2014) compared expertly vetted data obtained from various NHCs, many of which didn’t share data with GBIF, to unvetted data available from GBIF and, not surprisingly, found the unvetted data to be unreliable. They wrote “*Our results suggest that before conducting desktop assessments of the threatened status of species, aggregated museum locality data should be vetted against current taxonomy and localities should be verified. We conclude that available online databases are not an adequate substitute for taxonomic experts in assessing the threatened status of species and that Red List assessments may be compromised unless this extra step of verification is carried out*.” We agree. This study, and the consequent discussions on iPhylo and Taxacom covered many of the same concerns seen in Ferro and Flick (2015). These include issues such as how to best correct taxonomy and locality data, sharing of data, and georeferencing. One of the larger issues, which parallels Ferro and Flick (2015), is the lack of sharing of the expertly vetted data. Most of the NHCs from which the vetted data were obtained do not have sharing agreements with GBIF and the authors of Hjarding et al. (2015) did not share the vetted data. GBIF and similar data aggregators are not going to go away. They will improve with time but if those who have control of the highest quality data don’t share their data with GBIF, this improvement will be slow, to the detriment of all. The taxonomic community has the ability to overwhelm and replace any low-quality data in GBIF with data of the highest quality – to work together as part of the solution, rather than contribute to the problem. Researchers who work frequently with GBIF-mediated data often make suggestions for improvements to the error-reporting system itself. The GBIF community shares the desire to make it easy to report corrections and annotations in ways that the providers of the source data can see, handle and respond to. A key to making this happen is the wider adoption of consistent specimen and record level identifiers.

It is worth considering an analogy with GenBank regarding Ferro and Flick’s (2015) statement “*Online databases offer an opportunity for naïve or lethargic researchers to quickly produce poor quality research with little effort*.” Yes, a naïve user could download sequence data from a variety of genes for a small subset of the known species in a group, feed these into an automated alignment program and then without inspecting the alignment, generate a distance tree. The results would be a poor to worthless estimate of the group’s phylogeny. No one expects GenBank to warn users to prevent such poor science, and even less, no one would publish a critique of GenBank arguing GenBank is not to be trusted because it doesn’t have all genes for all species. Peer and editorial review are the gatekeepers that prevent poor science from being published. Reviewers and editors of work based on downloaded data from GBIF and other aggregators should be appropriately critical of authors’ methods. If an author was foolish enough to attempt to publish a niche model analysis of *Thoracophorus costalis* using only data from GBIF, we would hope that peer reviewers of such a manuscript would recommend rejection of the work, not because GBIF data were used, but rather because any reliable reviewer or editor should know that most entomological specimen data are not yet in GBIF. And conversely, if an author attempted to conduct a niche model analysis of this species and ignored the abundant and easily obtained high-quality data in GBIF for this species, we hope reviewers would require the authors to include the GBIF data or at least provide a rational justification for not doing so. Vetting GBIF data is now easier than before because of a new GBIF service that provides DOIs for any data download, which enables reviewers to easily examine the raw data on which analyses are based.

To carry the GenBank analogy a little further, imagine a researcher who assembled via their own lab-work a thorough genetic dataset to do a proper phylogenetic analysis of a taxon and then did the following (1) published a critique of GenBank complaining it lacked most of the data that the researcher had to generate and (2) held their data back rather than shared it with the scientific community. It is generally a requirement by journals for authors publishing on newly obtained genetic data to deposit their data with GenBank. It is our hope that the taxonomic community will see the benefits of treating specimen data the way most journals treat genetic data - as an investment in the greater good, as a way of raising the standards of taxonomic research, as a way of saving future generations the time and effort of digitizing specimens (again), as a way of making taxonomic research more useful for non-taxonomic researchers, and as a way of meeting the expectations of funding agencies. We need a GenBank for specimen data – a point made by Meier and Dikow (2004) who discuss the enormous potential value to conservation biology of the data published as part of taxonomic revisions.

## Biodiversity conservation

The taxonomic community is often quite vocal about conservation of biodiversity. Many conservation efforts are based on geo-political regions, be they nations, states, parks, or refuges. However, because taxonomy organizes data by taxon rather than region, it is easier to determine where a species occurs than to determine how many and which species occur in a region. For entomology, most of these data are found only on labels on pins scattered among various NHCs and scattered literature organized by taxon, not region. As a result, most regional checklists are usually limited in taxonomic scope (e.g. one large order or family).

If these data are shared globally they can be used for conservation of biodiversity related to land preservation or in analyses of shifting distributions resulting from climate change (e.g. Kerr et al. 2015). For example, to investigate the response of bumble bees to climate change, Kerr et al. (2015) were able to compile a georeferenced dataset for 67 species from Europe and North America that spanned 110 years. Records came from GBIF (171,479 North American and 192,039 European records), Bumblebees of North America (153,023 records), and the Status and Trends of European Pollinators Collaborative Project (237,586 records). These data came from institutions and organizations that digitized and shared their data. How many digitized and unshared records, or undigitized records that were not included in Kerr et al.’s analysis is unknown, but it is likely to be a very substantial number. We are headed towards a future in which specimen data that are not shared digitally will be increasingly overlooked. With the current re-evaluation of the Collections in Support of Biological Research (CSBR) program by the NSF, it is examples like this study of Kerr et al. (2015) that help illustrate the importance of NHCs to addressing big questions of global science. NHCs that refuse to (or are unable to) share their data will find themselves left out of such large collaborative studies and find it harder to justify future funding from programs like the NSF’s CSBR.

Any taxonomist who publishes new occurrence records but fails to share these data is, in effect, handicapping conservation efforts by hiding their taxa “in the dark” from geographically based searches. In particular, newly described species are often highly localized endemics known from few localities, or just the type locality. These species are of great interest to conservationists but it is the rarest of exceptions in entomology for occurrence data for these species to be shared with GBIF. Given the conservation importance of these species, and often the relatively few specimens involved, it is unfortunate that more such small and easily-prepared datasets are not shared.

Identifying and prioritizing more collections for digitization and publication through GBIF.org would serve the long-term needs of conservationists, while providing collections with greater visibility and return on investment because funding agencies are more likely to make awards to NHCs that are digitizing their holdings. A task force convened by GBIF is currently investigating how this can be best achieved through wide consultation with the global collections community (http://www.gbif.org/newsroom/news/accelerating-discovery-of-biocollections-data).

## Natural history collections digitization efforts

Imagine if all the NHCs from which Ferro and Flick (2015) had borrowed specimens had already databased and georeferenced their specimens and shared the data with GBIF? This would have reduced the time and cost of their study considerably. First, the task of verifying identifications would be easier. Most records shared with GBIF include the names of the determiners and the dates of determination, which enables evaluation of the trustworthiness of the records. Having a full dataset prior to borrowing any specimens would allow them to select only specimens that were identified by people that Ferro and Flick did not trust, were outliers in the distribution, or were needed for morphological study. This would reduce the number of loaned specimens and data capture efforts considerably. Incidentally, Ferro could improve the dataset by the correction of identifications. Secondly, while taxonomists are the best qualified to identify specimens they are not necessarily the best qualified to georeference specimens. Museum curators and collection managers who know the history, languages, and geography of the regions best represented in their collections, and the history of the collectors involved, can bring to bear far more knowledge for accurate georeferencing than can taxonomists who borrow specimens from various NHCs. A partnership between taxonomists and museums towards the creation of high-quality data is ideal.

## Conclusions and solutions

Historian J. J. O’Donnell, in his book Avatars of the Word (1998), notes the striking similarities between our current concerns about the internet (and digital data) and the responses of Medieval monks to the invention of the printing press. Their primary concern was that errors could creep into the bible and be duplicated hundreds of times, with no hope of gathering and destroying all erroneous copies. O’Donnell’s two main conclusions were (1) all technological change has consequences, good and bad, but (2) there is no stopping it. Ferro and Flick highlight the bad and provide useful caveats and warnings, but taxonomists should not turn their backs on this new reality. They should instead work to shape and improve it.

We welcome and appreciate the great effort invested by Ferro and Flick (2015) to compile and curate their dataset for *Thoracophorus costalis*. Coleopterists and other users of biodiversity data in modeling and research could benefit from wider access to such data. Working with scientific publishers, GBIF has strongly advocated for the broader use and acceptance of data papers as a means of gaining academic recognition of activities necessary for data collection, curation and publication (see http://www.gbif.org/mendeley/data-paper). We encourage Ferro to publish what is described as the “most comprehensive collection of distributional data for the species to date” (Ferro 2015), as a data paper (Costello et al. 2013, Costello and Wieczorek 2014). Datasets published through GBIF are automatically assigned a DOI and URL. Alternatively, in the spirit that any digital data are better than none, systematists can archive datasets with figshare.com or the Dryad Digital Repository (e.g. Sikes and Venables 2013a, b). Data in Dryad are not automatically shared with GBIF but are at least accessible openly for free download and use. Once permission is provided by each respective museum, a dataset like that produced by Ferro and Flick (2015), with records from various museums, can be archived directly in GBIF (e.g. Dikow 2012, Sikes and Mousseau 2013a, b). However, at present, GBIF and its participants only publish data from organizations — that is, institutions, networks, and societies — rather than individuals. Individuals wishing to publish data must work through their affiliated organizations or through journals (for example, Pensoft journals http://www.pensoft.net/journals), or GBIF nodes like Canadensys (http://www.canadensys.net/), which will publish data directly to GBIF from individual authors associated with a Canadian collection or organization (e.g. Schwarzfeld 2016). Ideally, such a service would be available to people from any nation, as GenBank is. It remains the case that many taxonomic organizations are not registered data providers, which is clearly a barrier to progress.

Because datasets generated from taxonomic revisionary work are the most thorough and high quality datasets available, we hope to see changes that enable these datasets to be more easily archived and shared. It is unrealistic and not efficient to expect all specimen digitization efforts to be performed by museums – especially when so much digitization is already being performed by taxonomists who borrow specimens. The changes necessary to realize this goal are both technological (e.g. easy access to data templates that can be filled in and user-friendly methods to share data) and behavioral (e.g. rewards for authors who take the extra effort to archive data, Chavan and Penev 2011). Scientific societies and journal editorial boards should encourage authors to deposit digital data. We direct readers interested in how to share data to a simple 10-step guide to data sharing written by Goodman et al. (2014) and the best-practices guide written by Costello and Wieczorek (2014). Dikow and Agosti (2015) recently published a valuable and relevant overview of new methods for sharing taxonomic data, with introduction of the term ‘cybercatalog’, and a description of Plazi, an effort to retroactively digitize taxonomic data by extracting it from legacy literature.

By publishing data papers and sharing their high-quality data, taxonomic experts critical of the quality of GBIF-mediated data can contribute constructively to improvements and at the same time gain wider visibility and recognition of their professional efforts. It has been asserted many times - the future of taxonomy is decline or digital renaissance (Godfray 2002, Maddison et al. 2012). Taxonomists and data aggregators should work together to maintain and advance the profile of biodiversity sciences. We know that to some this treatise probably sounds more like the Borg of Star Trek declaring “resistance is futile, prepare to be assimilated,” and that is the nature of O’Donnell’s conclusions. However, it need not be so bleak – GBIF is not the Borg, it is merely a data aggregator that helps users access data from various NHCs. We envision a bright future of well-maintained and well-digitized, growing Natural History Collections, and a thriving taxonomic community that continues to document our planet’s endless forms of most beautiful and wonderful life.
